# Renal Tumors in Pregnancy: A Case Report Focusing on the Timing of the Surgery and Patient Positioning

**DOI:** 10.1155/2022/1143478

**Published:** 2022-02-26

**Authors:** Hamidreza Ghorbani, Mahdi Mottaghi, Salman Soltani

**Affiliations:** Kidney Transplantation Complications Research Center, Mashhad University of Medical Sciences, Mashhad, Iran

## Abstract

Management of renal mass during pregnancy is challenging. There is no consensus regarding the fundamental timing issues (which trimester) of the interventions and patient positioning during the procedures. We present three pregnant women with renal mass and their management, focusing on patient positioning and timing of surgical intervention. All patients were positioned supine with a 30-degree rotation to the left lateral without signs of compromising fetal circulation. This report's three major takeaway points are the following: (1) Renal mass biopsy might be more beneficial in pregnant women than the normal population (unless CT findings suggest vascular angiomyolipomas) to achieve a definite diagnosis and avoid unnecessary interventions during pregnancy. (2) Surgical interventions, if indicated, should be performed as soon as possible and are applicable in all trimesters of pregnancy. (3) A minimum of 15-degree left lateral tilt (for both right- and left-sided renal masses) can provide enough venous return during the nephrectomy.

## 1. Introduction

Renal tumors are rare findings during pregnancy. Neoplasms complicate approximately 0.1% of all pregnancies; 0.013% are urological in origin [1]. Renal cell carcinoma (50%), angiomyolipoma (23%), and renal oncocytoma (3-7%) are the most frequently diagnosed renal tumors among pregnant women [2]. Renal cell carcinoma (RCC) is the most common tumor of the gestational period. It presents with flank mass, flank pain, hematuria, fever, and hypertension [3]. Management is the same as that of a nonpregnant patient [4]. Angiomyolipoma (AML) is the most common benign solid tumor of the kidneys overall and during pregnancy. Transarterial embolization (with precautions to minimize fetal radiation exposure) is the treatment of choice because it preserves renal function compared to nephrectomy. Oncocytomas are the most common benign enhancing kidney lesions [5]. They possess overlapping radiologic and histopathologic features with some subtypes of RCC (papillary and eosinophilic-chromophobe), and the diagnosis of oncocytoma is made after surgical resection in most cases. However, active surveillance is another option for biopsy-confirmed oncocytomas [6]. Metastatic lesions should be suspected if an extrarenal malignancy is present, and a renal mass biopsy is indicated to distinguish metastasis from infectious, inflammatory, hematologic, and primary renal tumors [9, 10].

Managing such tumors usually involves a nephrectomy (partial or complete). However, we found no disclosure and consensus on the optimal positioning of the pregnant woman during nephrectomy. Positioning should warrant sufficient venous return from the inferior vena cava to the heart, therefore enough cardiac output and placenta perfusion [7]. Herein, we aimed to report three pregnant women with kidney tumors and their management.

## 2. Case Presentation

### 2.1. Case 1

**Figure 1:**
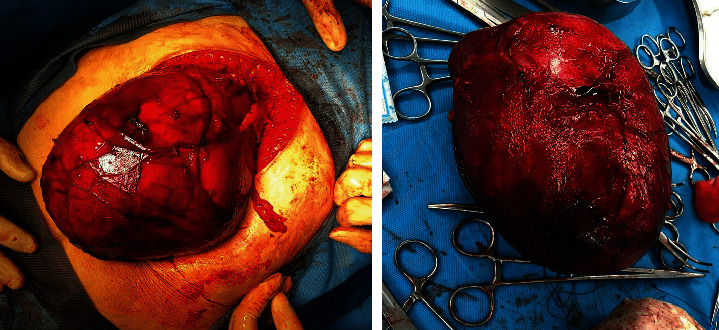
Macroscopic features of the clear cell RCC, resected with tumor-free margins.

A 32-year-old woman, gravida 2, para 1 (GA: 11 weeks), was presented with abdominal pain and heaviness more prominent in the left flank attributed to the pregnancy at first. However, the abdomen's progressive unequal distention in five weeks urged the patient to seek a further assessment. The physical exam was remarkable for a left flank mass. On the noncontrast MRI, a cystic multiloculated mass with numerous holes separated with thin septa was observed in the left kidney. Maximal dimension in the axial plane reported 191 mm. No solid components were seen. The radiologist suggested hydatid cyst strongly and cystic renal cell carcinoma (RCC) as another differential. After consultation with the gynecologist and infectious disease specialist, the consensus was obtained to perform a nephrectomy because of the rapid tumor growth. We performed the surgery at the 16^th^ week of gestation. The patient was positioned supine with a 30-degree rotation to the left lateral. Both the mother and baby were monitored during surgery via the nonstress test (NST) which remained stable during surgery. Macroscopically, a multicystic mass (80∗60∗15 mm) containing serous fluid was examined (Figure 1). Microscopically, several cysts with different sizes, fibrotic septa, simple cuboidal-columnar epithelium, bright (some acidophilic) cytoplasm, and nuclear atypia were detected. Cystic clear cell carcinoma was the final diagnosis with free surgical margins. Three years after the surgery, she is in a good state of health with no recurrence.

### 2.2. Case 2

A 24-year-old woman, gravida 2, para 1 (GA: 20 weeks), was presented with right flank pain. The Ultrasound (US) reported a hypoechoic hypervascular exophytic mass sized 50∗65∗110 in the right kidney's superior and middle pole. Mild pyelocaliceal distention was also reported. Color-Doppler US affirms the previous findings. MRI revealed a solid poor-fat renal tumor suggesting the RCC. After multidisciplinary consultation, radical nephrectomy was planned. The patient underwent an open nephrectomy at the 22^nd^ gestational week in the same position as the first case. For the macroscopic findings, a cream-colored mass sized 120∗90∗90 mm was examined. It was attached to the adrenal gland with no invasion. For the microscopic findings, a neoplastic growth composed of monomorphic spindle-cell proliferation with tortuous thick vasculature without necrosis and atypical was seen. Immunohistochemistry (IHC) studies were positive for actin, desmin, and HMB45 and negative for CD10, Ki57, CD117, CD34, S100, creatine kinase, and MELAN-A. These findings were consistent with angiomyolipoma of the kidney. She was healthy at her last follow-up visits with no complaints two years after surgery.

### 2.3. Case 3

**Figure 2:**
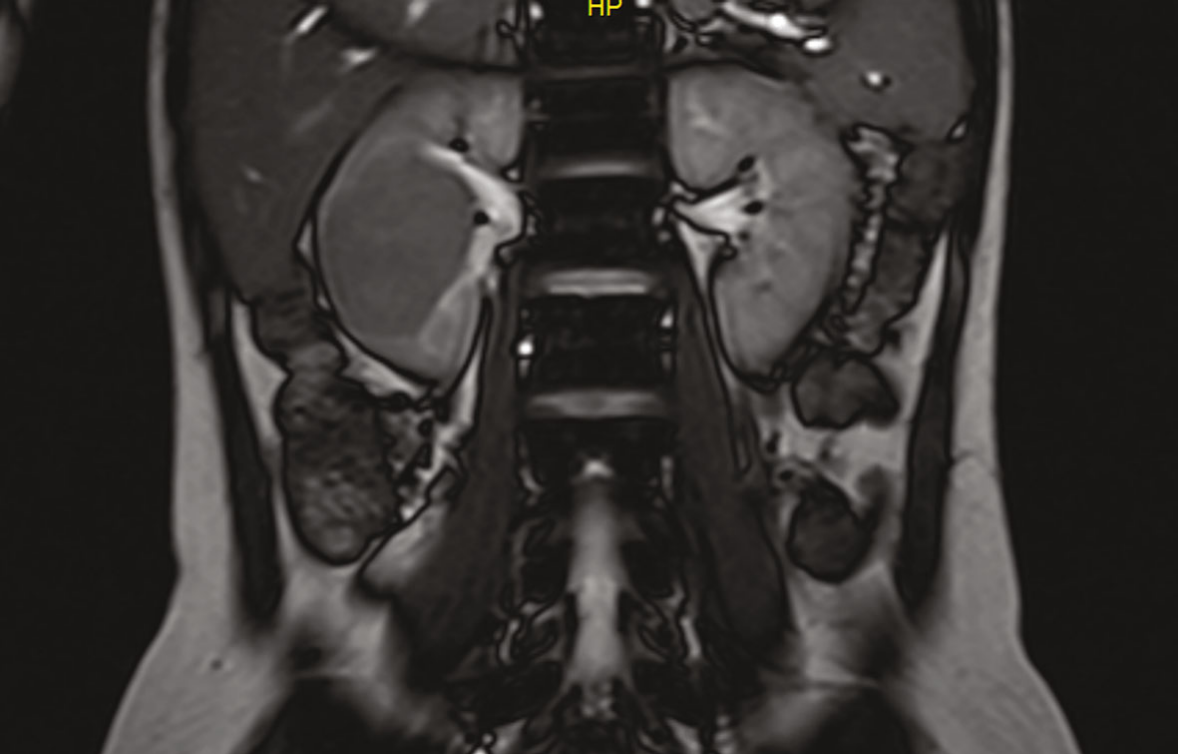
T2-weighted coronal section of the renal mass. Uterus containing fetus was visible in more anteriorly sections.

A 20-year-old primigravid woman (GA: 20 weeks) was referred to our tertiary clinic with right flank pain. The US reported a right renal mass of 60∗80∗40 mm. MRI showed an exophytic, low signal mass on T1 (Figure 2). T2-weighted images detected a low-intermediate signal of the tumor. A fine-needle biopsy was scheduled to minimize unnecessary surgery burden (by applying the previous case's experience). It confirmed the diagnosis of RCC, and we performed a total open nephrectomy at 23-week gestation. The surgery was conducted in the same position as the previous case. Macroscopic pathology reported a yellow tumor sized 50∗47∗45 mm with subcapsular extension plus a point capsular invasion but no vascular involvement. On microscopic evaluation, neoplastic cells with bright cytoplasm, mainly monomorphic, were seen. Foamy macrophages and foci of necrosis were detected between tubular and papillary structures (Fuhrman nuclear grade two). These findings were compatible with RCC with free surgical margins. IHC was not performed. She got pregnant with her second child 21 months later and had no clinical and radiological evidence of recurrence.

## 3. Discussion

Management of renal mass during pregnancy is a challenging topic. There is no consensus regarding the fundamental timing issues (which trimester) of the interventions and patient positioning during the procedures. All solid renal tumors during pregnancy should be considered RCC unless proven otherwise. To rule out this possibility, a timely nephrectomy (partial or radical) should be performed to make a definite diagnosis. Ohyama et al. presented a case of renal mass in pregnancy in which the diagnosis was confirmed preoperatively via fine-needle biopsy; however, one should remember that the negative results do not rule out malignancy [8].

### 3.1. Patient Positioning

The kidney is best accessible when the patient is positioned lateral decubitus on a flexed table. Table flexion may increase the uterus' pressure; thus, we suggest a straight table. Therefore, a tilted table to the right or left can reduce this effect. Here, we proceed to review two possible positions for the nephrectomy of a pregnant woman. Of note, these positions started with the adjective “relative” to show their difference with full left and right decubitus positions.

#### 3.1.1. Relative Left Lateral Position (RLLP)

At the second and third trimesters, aortocaval compression can cause hypotension and impaired placental perfusion [9]. It can be avoided via an RLLP with at least 15-45-degree tilt for right kidney tumors [10–12]. This position provides optimum venous return even during a pregnant woman's cardiopulmonary resuscitation with one resuscitator [13]. Less than 15-degree tilt cannot provide additional benefits than a pure supine position [10]. We performed all nephrectomies in this position, and none of our patients experienced hemodynamic instability during the procedure and the following 24 hours.

#### 3.1.2. Relative Right Lateral Position (RRLP)

A recent study on ten pregnant women showed a 35% lower venous return in RRLP than RLLP; however, this difference in cardiac output and blood flow of the abdominal aorta (and thus the placental perfusion) is insignificant between left and right lateral decubitus positions [14]. Fujita et al. analyzed thirteen pregnant women and concluded that IVC volume is most at a 30-degree tilt to the left and right in 70% and 23% of patients, respectively [10]. Julien et al. performed a diaphragmatic hernia repair while the pregnant patient was in the RRLP. They simulate the position preoperatively with fetal monitoring to ensure fetal and maternal hemodynamic stability [15].

### 3.2. Timing of Intervention

RCC is the most common tumor in pregnancy and seems heterogeneous; each patient showed variable tumor size, manifestation, and growth rate [8]. A meta-analysis of 286 solid localized renal lesions revealed a mean growth rate of 0.28 cm each year (median 0.28, range 0.09 to 0.86) during a mean follow-up of 34 months (median 32, range 26 to 39) [16]. Tumors with higher growth rates should be managed with prompt interventions. Delayed intervention can result in devastating outcomes. Bettez et al. reported a renal tumor during pregnancy with MRI findings suggestive of an angiomyolipoma [17]. They postponed the surgery until only three days after delivery, where CT found an invading renal mass, which was managed with subsequent nephrectomy. The patient was diagnosed with clear cell RCC. After three months, follow-up CT revealed multiple distant metastases, and the patient died after 12 months [17]. Inversely, Akpayak et al. postponed a renal tumor surgery based on the mother's wishes until after delivery [18]. The patient underwent surgery after delivery and was diagnosed with clear cell RCC similar to the previous case; however, no metastasis was found during follow-up. The only difference between these cases is the tumor growth rate, which was higher in the first case.

Although interventions during the first trimester have an increased risk of spontaneous abortion, we recommend surgery unless there is a previous infertility history [8]. In case of a history of infertility, the decision to perform surgery needs extra caution, and delayed intervention until the second trimester is another option provided close follow-up of the tumor growth rate. The second trimester is the best time of surgery suggested by many authors with the least danger to the fetus.

Surgical interventions for renal tumors diagnosed in the third trimester (after 28 weeks of the last menstrual period) can be delayed after fetal lung maturation in the presence of reliable patient follow-up and the absence of a concerning growth rate. Typically, lung maturation is completed enough for breathing out of the mother's body after the 35^th^ week of gestation; however, this time limit can be brought forward to the 28^th^ week of pregnancy (lung maturation point via steroids) [2, 8, 19]. Simultaneous caesarian section and nephrectomy (partial or complete) can be performed, but urologists and gynecologists need close cooperation to control blood loss during surgery and postoperative complications [18].

### 3.3. Complication

Venous thromboembolism and abortion are two major reported complications of RCCs during pregnancy [17, 20]. Fortunately, our patients experienced no complications.

### 3.4. Prevention

Women of reproductive age who have planned to conceive should consider kidney imaging before pregnancy [21].

## 4. Conclusion

This report's three takeaway notes are the following: (1) Biopsy should be considered (unless CT findings suggest vascular angiomyolipomas) to achieve a definite diagnosis and avoid unnecessary interventions during pregnancy. (2) Surgical interventions, if indicated, should be performed as soon as possible and are applicable in all trimesters of pregnancy. (3) A minimum of 15-degree left lateral tilt (for right-sided renal mass) and 30-degree right lateral angle (for left-sided renal tumor) can provide enough venous return during the nephrectomy.

## Data Availability

Data used to write this case report is accessible through contact with the corresponding author, Soltani S, email: soltanis@mums.ac.ir.
